# Influence of microbially fermented 2´-fucosyllactose on neuronal-like cell activity in an *in vitro* co-culture system

**DOI:** 10.3389/fnut.2024.1351433

**Published:** 2024-02-08

**Authors:** Sabine Kuntz, Clemens Kunz, Christian Borsch, David Hill, Sinéad Morrin, Rachael Buck, Silvia Rudloff

**Affiliations:** ^1^Department of Nutritional Science, Justus Liebig University Giessen, Giessen, Germany; ^2^Abbott, Nutrition Division, Columbus, OH, United States; ^3^Department of Pediatrics, Justus Liebig University Giessen, Giessen, Germany

**Keywords:** 2´-fucosyllactose, fermentation, microorganisms, neuronal-like cell activity, BDNF

## Abstract

**Scope:**

2´-Fucosyllactose (2´-FL), the most abundant oligosaccharide in human milk, plays an important role in numerous biological functions, including improved learning. It is not clear, however, whether 2´-FL or a cleavage product could influence neuronal cell activity. Thus, we investigated the effects of 2´-FL, its monosaccharide fucose (Fuc), and microbial fermented 2´-FL and Fuc on the parameters of neuronal cell activity in an intestinal–neuronal transwell co-culture system *in vitro*.

**Methods:**

Native ^13^C-labeled 2´-FL and ^13^C-Fuc or their metabolites, fermented with *Bifidobacterium (B.) longum* ssp*. infantis* and *B. breve*, which were taken from the lag-, log- and stationary (stat-) growth phases of batch cultures, were applied to the apical compartment of the co-culture system with Caco-2 cells representing the intestinal layer and all-*trans*-retinoic acid-differentiated SH-SY5Y (SH-SY5Y_ATRA_) cells mimicking neuronal-like cells. After 3 h of incubation, the culture medium in the basal compartment was monitored for ^13^C enrichment by using elemental analysis isotope-ratio mass spectrometry (EA-IRMS) and effects on cell viability, plasma, and mitochondrial membrane potential. The neurotransmitter activation (BDNF, GABA, choline, and glutamate) of SH-SY5Y_ATRA_ cells was also determined. Furthermore, these effects were also measured by the direct application of ^13^C-2´-FL and ^13^C-Fuc to SH-SY5Y_ATRA_ cells.

**Results:**

While no effects on neuronal-like cell activities were observed after intact 2´-FL or Fuc was incubated with SH-SY5Y_ATRA_ cells, supernatants from the stat-growth phase of 2´-FL, fermented by *B. longum* ssp. *infantis* alone and together with *B. breve*, significantly induced BDNF release from SH-SY5Y_ATRA_ cells. No such effects were found for 2´-FL, Fuc, or their fermentation products from *B. breve*. The BDNF release occurred from an enhanced vesicular release, which was confirmed by the use of the Ca^2+^-channel blocker verapamil. Concomitant with this event, ^13^C enrichment was also observed in the basal compartment when supernatants from the stat-growth phase of fermentation by *B. longum* ssp. *infantis* alone or together with *B. breve* were used.

**Conclusion:**

The results obtained in this study suggest that microbial products of 2´-FL rather than the oligosaccharide itself may influence neuronal cell activities.

## 1 Introduction

Human milk oligosaccharides (HMOs) are the third largest solid component in human milk, present to the extent of 20–25 g/L in colostrum and 10–15 g/L in mature milk (1–5). 2´-Fucosyllactose (2´-FL) belongs to the fraction of fucosylated neutral HMOs and is quantitatively the most prominent component in the breastmilk of women expressing fucosyltransferase-2 (FUT-2), a phenotype referred to as secretor positive and representing 70–80% of the Western population (3–8). 2´-FL is a well-known structural homolog to bacterial adhesion sites in the intestine and may act as a prebiotic, supporting colonization of the colon with bacteria that may be beneficial to the breastfed infant (9–11).

In infants, breast milk feeding is known to provide significant health benefits and may even improve cognitive development and intellectual performance (12–16). In this context, 2´-FL or Fuc has been shown to affect cognitive domains and improve learning and memory in animal studies (17–19). 2´-FL is also associated with improved cognition (18, 20) and changes in brain tissue microstructure in breastfed infants (21). The mechanisms behind these neuronal effects are largely unknown. For example, a continuous administration of 2´-FL increased the expression of several molecules involved in the storage of newly acquired memories, such as the postsynaptic density protein 95, phosphorylated calcium/calmodulin-dependent kinase II, and brain-derived neurotrophic factor (BDNF) in cortical and subcortical structures (17). BDNF and its isoforms are members of the neurotrophin family and are synthesized by both, neuronal and non-neuronal cells. They are involved in processes such as differentiation and regeneration (22, 23). It has also been shown that BDNF plays a key role as a mediator of activity-induced long-term potentiation (LTP) in the hippocampus as well as in other brain regions (24). The role of BDNF and its isoforms in LTP is best studied in the hippocampus where the neurotrophins act at pre- and post-synaptic levels and are mediated by Trk (tropomyosin-related kinase) and the tumor necrosis factor receptor family, which are known to be coupled to the activation of the Ras/ERK, phosphatidylinositol-3-kinase/Akt and phospholipase C-g(PLC-g) pathways, and proBDNF/p75NTR/sortilin pathways (24–26). In addition, BDNF is the most important modulator of glutamatergic and GABAergic synapses and is also associated with glutamate and GABA through TrkB signaling (24, 27).

However, it is unclear whether 2´-FL itself or its metabolites are responsible for the observed effects. To achieve neuronal effects, 2´-FL or its metabolites may need to accumulate in the relevant brain regions; however, as we have recently shown, ^13^C-labeled 2´-FL administered orally to wild-type and germ-free mice was unable to cross the healthy blood–brain barrier (28). A subsequent study showed that even the Fuc moiety from 2´-FL, administered as ^13^C-labeled Fuc, was also not able to cross the blood–brain barrier either, although it was rapidly absorbed. It was observed that ^13^C was enriched in the brain at time points after the oral bolus had reached the lower gut (29). This points to the influence of the intestinal microbiota, which are shown to metabolize HMOs and selectively promote the growth of beneficial microbiota such as bifidobacteria (30, 31). Metabolic studies in infants have demonstrated that 2´-FL in milk from secretor mothers is excreted via infants´ urine (32), which was confirmed by endogenously ^13^C-labeled HMOs in breastfeeding mothers and the urinary excretion of ^13^C-labeled HMO in their infants (33–36). Low amounts of 2´-FL have also been detected directly in the plasma of breastfed infants of secretor mothers compared to infants fed milk from non-secretor mothers or in plasma from formula-fed infants only when 2’FL was added as a supplement (37, 38). Despite the absorption of intact 2´-FL into the circulation, HMOs are not digested by human enzymes and reach the colon where they are metabolized by the infant gut microbiota.

In general, HMOs are substrates for beneficial microbes such as species of the *Bifidobacterium* genus, but it seems that only a few strains use HMOs as a preferred carbon source (39–43). However, the uptake of HMOs by microbial ABC transporters and their degradation by glycosyl hydrolases result in the formation of monosaccharides, which could be further metabolized by the fructose-6-phosphate phosphoketolase pathway into ATP, acetate, and lactate as end products, which was observed in the case of *B. longum* ssp. *infantis* (44–47). In contrast, extracellular glycosyl hydrolases of *B. breve* and *B. bifidum* generate metabolites that may serve as substrates for *B. longum* ssp. *infantis*, which highlights the co-existing or cross-feeding effects influencing HMO metabolism (48, 49). This microbe–HMO interaction was supported by an accumulation of HMO building blocks such as Fuc and trisaccharides after fermentation of HMO by bifidobacteria and lactobacilli, suggesting a symbiotic interaction of HMOs and specific gut microbiota (50). Recently, the analysis of the development of the gut microbiota of infants during the first month of life shows that colonization of FL-utilizing *Bifidobacteria* species is associated with altered metabolite profiles and microbiota composition (44). Equal co-cultures of bifidobacteria in 2´-FL-containing media produced different ratios of metabolites such as acetate and lactate under steady-state conditions when compared to monocultures (45). Furthermore, it has recently been confirmed that HMOs such as 2´-FL selectively promote the formation of a bifidobacteria-rich microbiota (30), which may then increase their potential impact on neurological functions via the gut–brain axis.

The overall aim of our *in vitro* intestinal–neuronal transwell co-culture system was to investigate if and how ^13^C-labeled 2´-FL as well as its monosaccharide Fuc were metabolized by different *Bifidobacterium* species, alone or in combination, and if intact or subsequent metabolites cross the monolayers of Caco-2 cells cultured on transwell inserts to affect neuronal-like parameters in neuronal-like ATRA-differentiated SH-SY5Y_ATRA_ cells.

## 2 Results

### 2.1 Effects of 2´-FL and Fuc on neurogenesis markers in neuronal-like cells before and after passage through an intestinal epithelial cell layer

**Figure 1:**
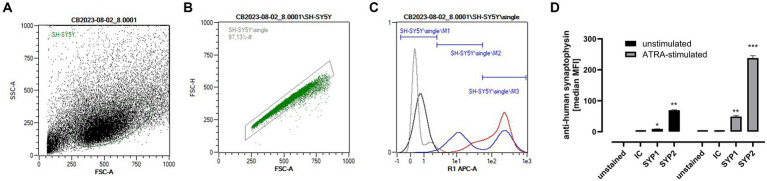
Synaptophysin (SYP) expression in SH-SY5Y and ATRA-differentiated SH-SY5Y cells. Differentiation of SH-SY5Y to SH-SY5Y_ATRA_ was confirmed by measuring SYP expression by flow cytometry. Differentiated and non-differentiated cells were detached with accutase solution, centrifugated (500 × *g*, 5 min at RT) and stained according to the manufactures´ instructions (see Methods and Materials 4.1.2). The gating strategy for analyzing SYP expression is given for a representative staining in **(A**–**C)**. [**(A)** dotblot for cell gating (SSC-A vs. FSC-A), **(B)** dotblot for single cell inclusion (FSC-H vs. FSC-A), and **(C)** representative histogram of unstained cells (gray line), isotype control (IC) stained cells (black line), undifferentiated anti-human-SYP stained cells (blue line), and anti-human-SYP stained ATRA-induced cells (red line)]. **(D)** Quantification of the MFI (mean fluorescence intensity) was performed by setting histogram markers (M) for unstained and IC-stained cells (M1), low SYP (SYP1) expressing cells (M2), and high SYP (SYP2) expressing cells (M3). MFI data were performed using the MACSQuant 2.13.0 software and data analyses (medians with 95% CI) were performed with GraphPad Prism 10.0.2. Differences to IC-stained cells were significant at *p < 0.05, **p < 0.01, and ***p < 0.05 (ANOVA with multicomparison test) for at least *n* = 3 (in duplicates).

To investigate the effects of 2´-FL and Fuc on neuronal-like cell activity markers, we used the human cell line SH-SY5Y, which had been differentiated by all-*trans*-retinoic acid (ATRA) into cells with a significant expression of the well-known neuronal marker synaptophysin (SYP) (51, 52) determined by flow cytometry (Figures 1A–C). Figure 1D shows that in the cultured SH-SY5Y cells, cell populations with both low and high SYP expression levels were present (Figure 1D). Incubation of these SH-SY5Y cells with ATRA over 10 days induced a significant enhancement of cells with high SYP expression, which is 2.6 times higher than in unstimulated cells.

**Figure 2:**
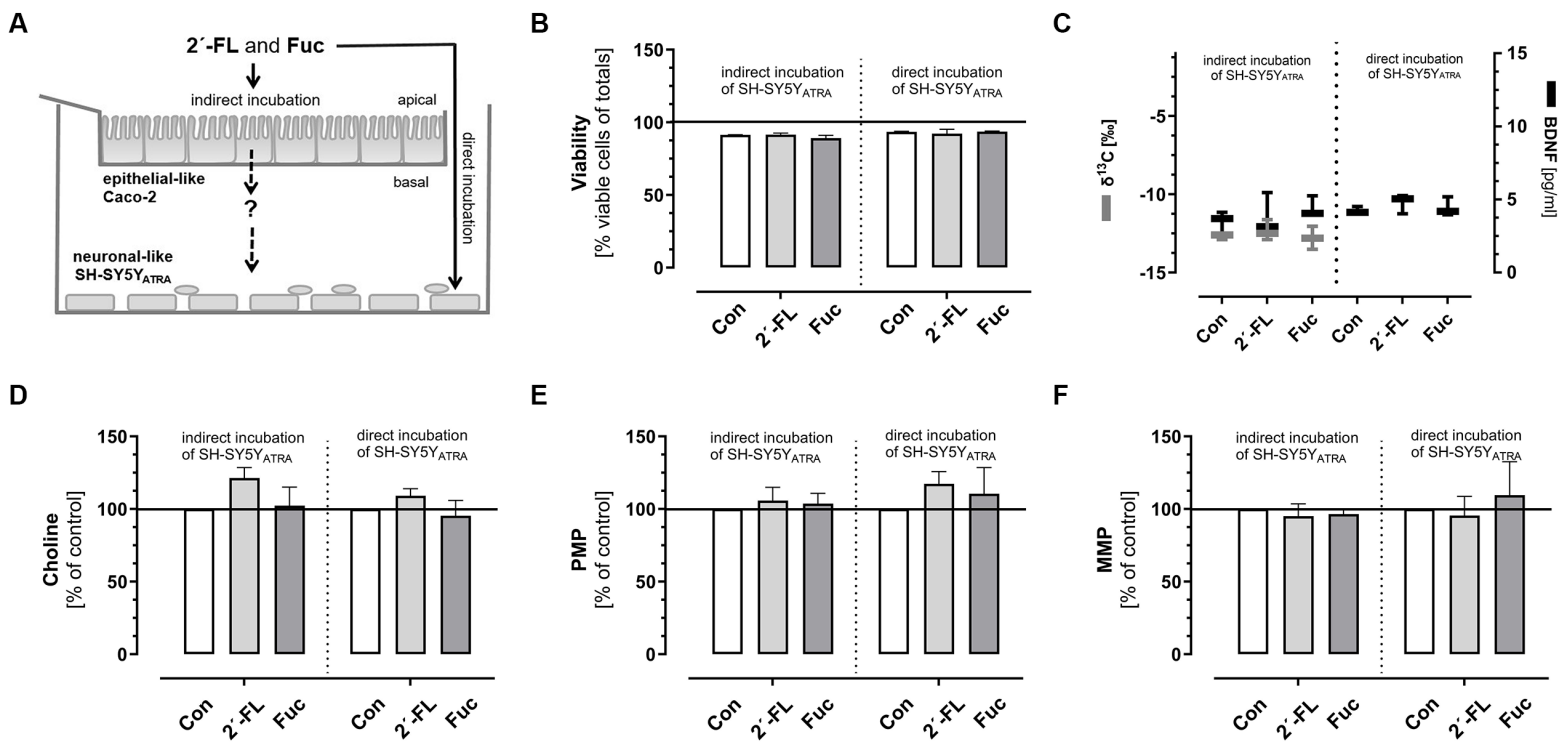
Determination of indirect and direct effects of 2´-FL and Fuc on ATRA-differentiated neuronal-like SH-SY5Y cells (SH-SY5Y_ATRA_). Caco-2 cells, cultured on transwell inserts, were incubated with 2´-FL or Fuc (5 mM) for 3 h at 37°C. Thereafter, transwell inserts were removed and SH-SY5Y_ATRA_ cells were further incubated with basal media (indirect incubation) or directly with 2´-FL or Fuc (0.5 mM) in comparison to controls (5 mM Glucose) **(A)**. Viability was measured by flow cytometry using the ViaCount Reagent^®^ [% viable cells of total cells] **(B)**, ^13^C enrichment [δ^13^C in ^0^/_00_] was determined by EA-IRMS [**(C)**, left *Y*-axis], BDNF concentrations [pg/mL] were measured in the supernatant by ELISA [**(C)**, right *Y*-axis], choline levels **(D)**, plasma membrane potential (PMP) and mitochondrial membrane potential (MMP) were determined fluorometrically and are given as % of controls **(E,F)**. Data are shown as box blots with min-max (whiskers) or as bars with means and standard deviation for *n* = 3 (each in duplicate). Significant differences were calculated by *t*-test comparing control with 2´-FL or Fuc.

These neuronal-like SH-SY5Y_ATRA_ cells were used to investigate the effects of 2´-FL and Fuc with or without co-cultured Caco-2 cells (Figure 2A). Therefore, 2´-FL and Fuc were applied at non-cytotoxic concentrations (Figure 2B) to the apical side of the transwell (indirect incubation) or directly to SH-SY5Y_ATRA_ cells. As shown in Figures 2C–F, incubation with ^13^C-2´-FL and ^13^C-Fuc (5 mM) at the apical side of the co-culture system did not result in any ^13^C enrichment [δ^13^C in ^0^/_00_] in the basal compartments (Figure 2C, left *Y*-axis) compared to controls (5 mM glucose), nor did it induce BDNF release (Figure 2C, right *Y*-axis) in the supernatant or choline levels in the cells (Figure 2D). Consistent with these results, no changes in plasma membrane or mitochondrial potential were observed by direct or indirect incubation with 2´-FL or Fuc (Figures 2E,F).

These observations clearly indicate that neither 2´-FL nor Fuc had an influence on neuronal activity markers when they were applied to neuronal-like cells directly or indirectly. Due to the low concentration of intact 2´-FL or Fuc in systemic circulation and recently published data about the intense fermentation of 2´-FL and Fuc in the intestine of mice (28, 29), we aimed to investigate whether the fermentation of 2´-FL and/or Fuc by *Bifidobacterium* species had an influence on neuronal cell activity markers. Again, we used ^13^C-labeled 2´-FL as well as Fuc. To gain further insight into the metabolic pathways of 2´-FL and/or Fuc during microbial fermentation, we used 2´-FL and Fuc either ^13^C-labeled on C-atom 1 (^13^C_1_-Fuc) or 6 (^13^C_6_-Fuc).

### 2.2 Microbial fermentation of 2´-FL and Fuc

**Figure 3:**
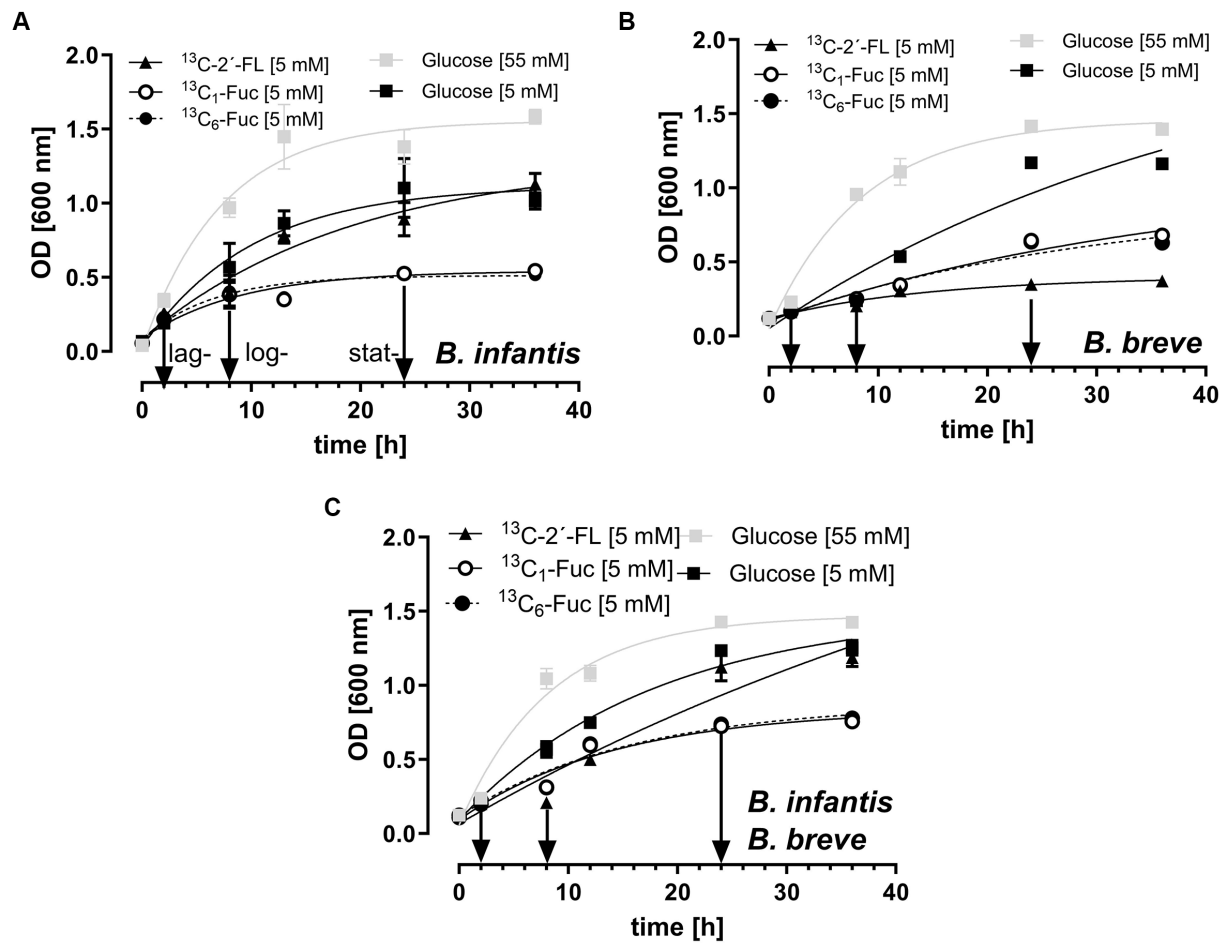
Growth of *B. longum* ssp*. infantis* **(A)**, *B. breve* **(B)**, and co-culture of *B. longum* ssp*. infantis with B. breve* **(C)** in high glucose-containing medium (55 mM), glucose-reduced media (5 mM), ^13^C-2´-FL- and ^13^C-Fuc-supplemented media (5 mM). Bacterial strains were anaerobically cultured at 37°C (see Methods and Materials 4.1.5) and growth was measured spectrophotometrically (600 nm). Data are given as means and standard deviation for *n* = 3. Arrows indicate the collection time of growth media at lag-, log- and stat-growth phase of the batch cultures.

For fermentation studies, we used *B. longum* ssp*. infantis* and *B. breve* as bifidobacterial strains, as they are known to ferment HMOs by extra- and intracellular glycosyl hydrolases and have the potential for bifidobacterial cross-feeding (50). As shown in Figures 3A–C, all the bacterial strains grew well in media containing high concentrations of glucose (55 mM). *B. longum* ssp*. infantis*, *B. breve*, and co-cultured bifidobacteria grew rapidly and reached an optical density (OD_600 nm_) values of 1.58 ± 0.05, 1.39 ± 0.03, and 1.41 ± 0.05, respectively. When these strains were grown in media containing 5 mM glucose instead of 55 mM glucose, they still grew well, but with a lower maximum OD_600 nm_ values of 1.02 ± 0.07, 1.16 ± 0.05, and 1.25 ± 0.04 after 36 h of incubation, respectively (Figures 3A–C). Substitution of this lower glucose concentration of 5 mM with an isomolar concentration of 2´-FL as the sole carbohydrate source, *B. longum* ssp*. infantis* alone (Figure 3A) or in co-culture with *B. breve* (Figure 3C) grew to an optical density (OD_600 nm_) similar to that with 5 mM glucose (1.12 ± 0.07 and 1.16 ± 0.06). However, in media containing ^13^C_1_-Fuc- or ^13^C_6_-Fuc, *B. longum* ssp*. infantis* grew very slowly with maximum OD_600 nm_ values of 0.54 ± 0.02 and 0.52 ± 0.01. In contrast, *B. breve* showed better growth on ^13^C_1_-Fuc and ^13^C_6_-Fuc-containing media with a maximum optical density of 0.68 ± 0.01 and 0.63 ± 0.01, respectively, but did not grow in media containing ^13^C-2´-FL as a carbohydrate source (Figure 3B).

**Figure 4:**
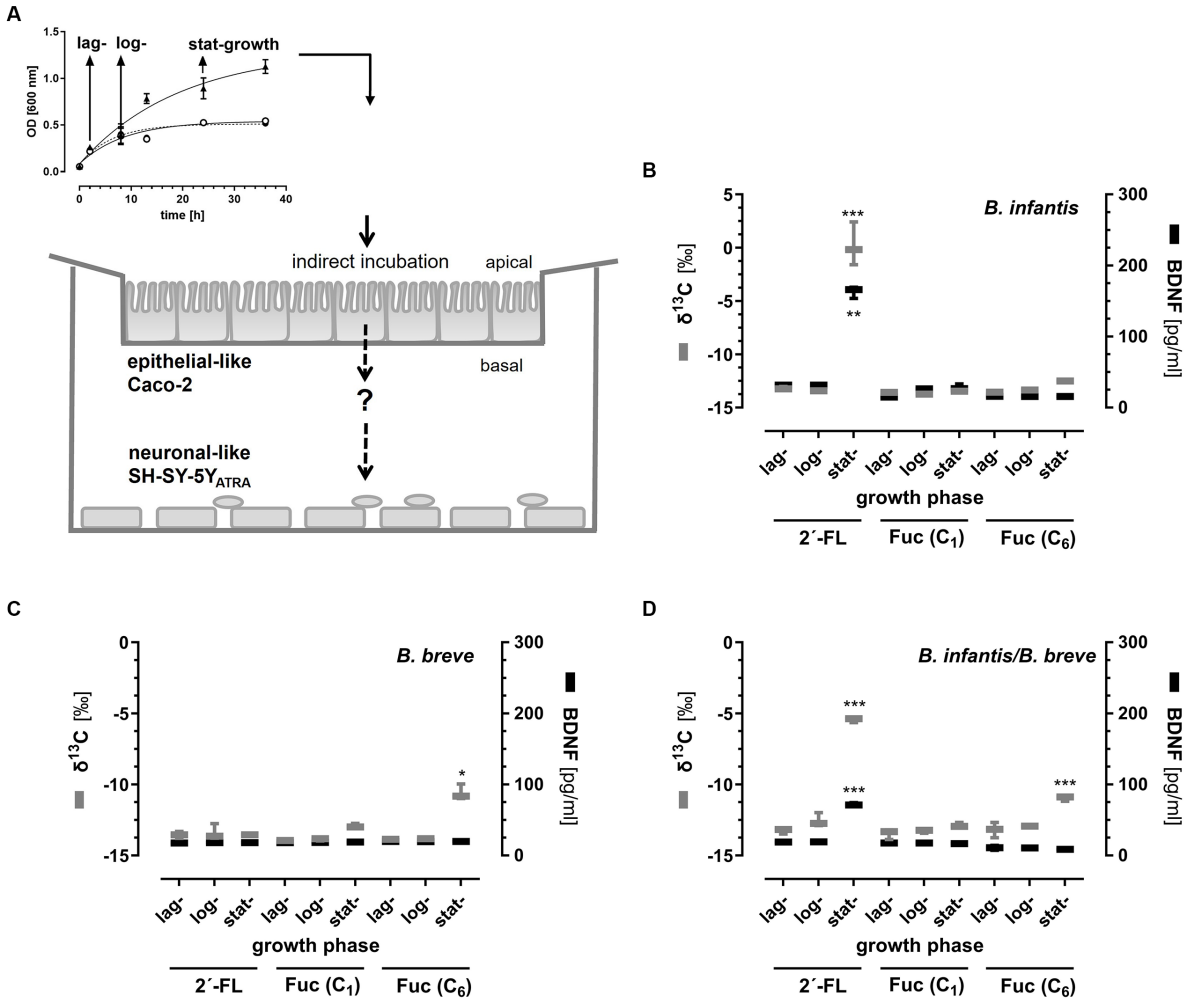
Enrichment of ^13^C-2´-FL- and ^13^C-Fuc-derived metabolites in the basal compartments of a transwell system and their influence on BDNF-secretion from neuronal-like SH-SY5Y_ATRA_ cells. *B. longum* ssp*. infantis, B. breve*, and *B longum* ssp. *infantis* together with *B. breve* were grown in ^13^C-2´-FL-, ^13^C-Fuc (^13^C_1_-Fuc or ^13^C_6_-Fuc)-containing (5 mM) media. At the lag -, log-, and stat-growth phase, growth media were collected, centrifuged, filtered, and pH-adjusted (pH 7.4). Thereafter, supernatants were applied to the transwell inserts containing the Caco-2 cell monolayer (apical) and neuronal-like SH-SY5Y_ATRA_ cells (basal) **(A)**. In the first set of experiments, basal compartments were collected after a 3 h incubation of Caco-2 cells to determine the ^13^C enrichment by EA-IRMS being expressed as δ^13^C [^0^/_00_] [**(B–D)** left *Y*-axis]. In a second set of experiments, SH-SY5Y_ATRA_ cells were further incubated with basal media for 21 h to measure BDNF secretion by ELISA given as pg./mL [**(B–D)** right *Y*-axis]. Data are shown as box blots with min-max (whiskers) with n = 3 (each in duplicate). Significant differences between lag-, log-, and stat-growth phase values were calculated with one-way ANOVA with multi-comparison tests. Differences were significant at *p < 0.05, **p < 0.01, and ***p < 0.001.

To investigate the possible effects of fermentation products on neuronal cell activity makers, we collected growth media from 2´-FL- and Fuc-fermented batch cultures at three different time-points: lag-, log- and stat-growth phase. The collected supernatants were used in the intestinal–neuronal transwell co-culture system (Figure 4A).

### 2.3 Effects of bacterial fermentation products on SH-SY5Y_ATRA_ cells in a co-culture model

In the first set of experiments, we aimed to investigate whether bacterial fermentation products collected at the three different time points during batch cultures passed an intestinal Caco-2 cell monolayer and reached the basal compartment of the transwell system. In the second set of experiments, we measured BDNF secretion from SH-SY5Y_ATRA_ cells after a 24 h incubation with the enriched basal media (Figures 4B–D).

Using cell-free media from different time points of bacterial growth, we observed significant ^13^C enrichment and a concomitant BDNF secretion only with stat-growth phase 2´-FL metabolites from *B. longum* ssp*. infantis* in the basal compartments (Figure 4B) and, to a lesser extent, with 2´-FL metabolites from the stat-growth phase of *B. longum* ssp*. infantis* co-cultured with *B. breve* (Figure 4D). In the *B. breve* cultures, neither ^13^C enrichment nor BDNF secretion by SH-SY5Y_ATRA_ cells was observed with fermented 2´-FL metabolites (Figure 4C). However, when *B. breve* was incubated with ^13^C-Fuc, ^13^C enrichment was observed after the fermentation of Fuc (lag-growth phase) when C_6_ atom of Fuc was ^13^C-labeled, but not when ^13^C_1_-Fuc was used. This was also observed when *B. breve* was *co*-cultured with *B. longum* ssp*. infantis.* Interestingly, no secretion of BDNF was observed by SH-SY5Y_ATRA_ cells when Fuc metabolites were present in the basal media (Figures 4B–D).

In addition to BDNF, we could not detect any further effect on other potential neurotransmitters such as GABA (γ-aminobutyric acid) or the precursor molecule glutamate (see Supplementary Figures S1, S2). Further, it should be noted that the secretion of BDNF by differentiated neuronal-like cells was relatively low. Thus, the secreted amounts of BDNF in the co-culture system may not have been sufficient to influence further neurotransmitter release.

### 2.4 Effect of the calcium channel blocker verapamil on BDNF secretion from differentiated SH-SY5Y_ATRA_ cells after incubation with bifidobacterial fermentation products

**Figure 5:**
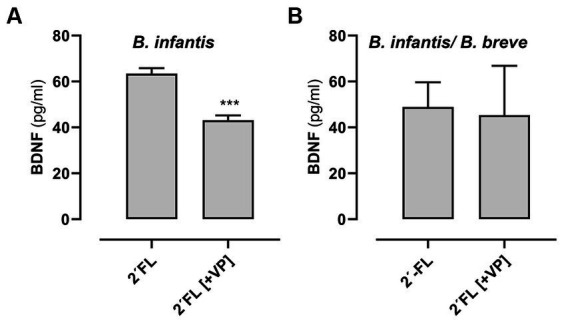
Effect of the calcium channel blocker verapamil (VP) on BDNF secretion from neuronal-like SH-SY5Y_ATRA_ cells after incubation with stat-growth phase metabolite-enriched media. Neuronal-like SH-SY5Y_ATRA_ cells were pre-incubated with 7.5 μM VP for 30 min or without VP. Thereafter, the cells were incubated for 24 h with the metabolite-enriched medium from stat-growth phase from *B. longum* ssp*. Infantis* **(A)** and *B. longum* ssp. Infantis co-cultured with *B. breve* **(B)**. After incubation with enriched media, BDNF secretion [pg/mL] was measured in the supernatant using ELISA (see Methods and Materials 4.4 and 4.9). Data are given as means ± standard deviation for *n* = 3 (in duplicates). Differences between cells with and without VP-treatment were analyzed with *t*-test and were significant at ***p < 0.001.

Based on the results with the stat-growth phase 2´-FL metabolites from *B. longum* ssp. *infantis* alone or grown together with *B. breve* on BDNF secretion by neuronal-like SH-SY5Y_ATRA_ cells, we probed further with the aim of understanding the mechanism of the enhanced secretion. This secretion could be a result of increased mRNA expression or the release from secretory vesicles (53, 54). Therefore, we used Verapamil (VP) as a L-type calcium channel blocker to verify the effects on vesicular release and additionally RT-qPCR to measure mRNA expression. As shown in Figures 5A,B, BDNF release induced by 2´-FL metabolites from *B. longum* ssp*. infantis* was partially reduced by pre-incubation of SH-SY5Y_ATRA_ cells with VP (Figure 5A). This effect was not observed for 2´-FL metabolites generated by *B. longum* ssp*. infantis* co-cultured with *B. breve* (Figure 5B). Due to the incomplete inhibition by VP, other calcium channels (e.g., N-, T-type) may also play a role. In contrast to the inhibiting effect of VP on BDNF release by *B. longum* ssp*. infantis*, we did not observe any changes in mRNA expression due to 2´-FL metabolites produced by *B. longum* ssp*. infantis* nor by *B. longum* ssp*. infantis co*-cultured with *B. breve* (see Supplementary Table T1).

**Figure 6:**
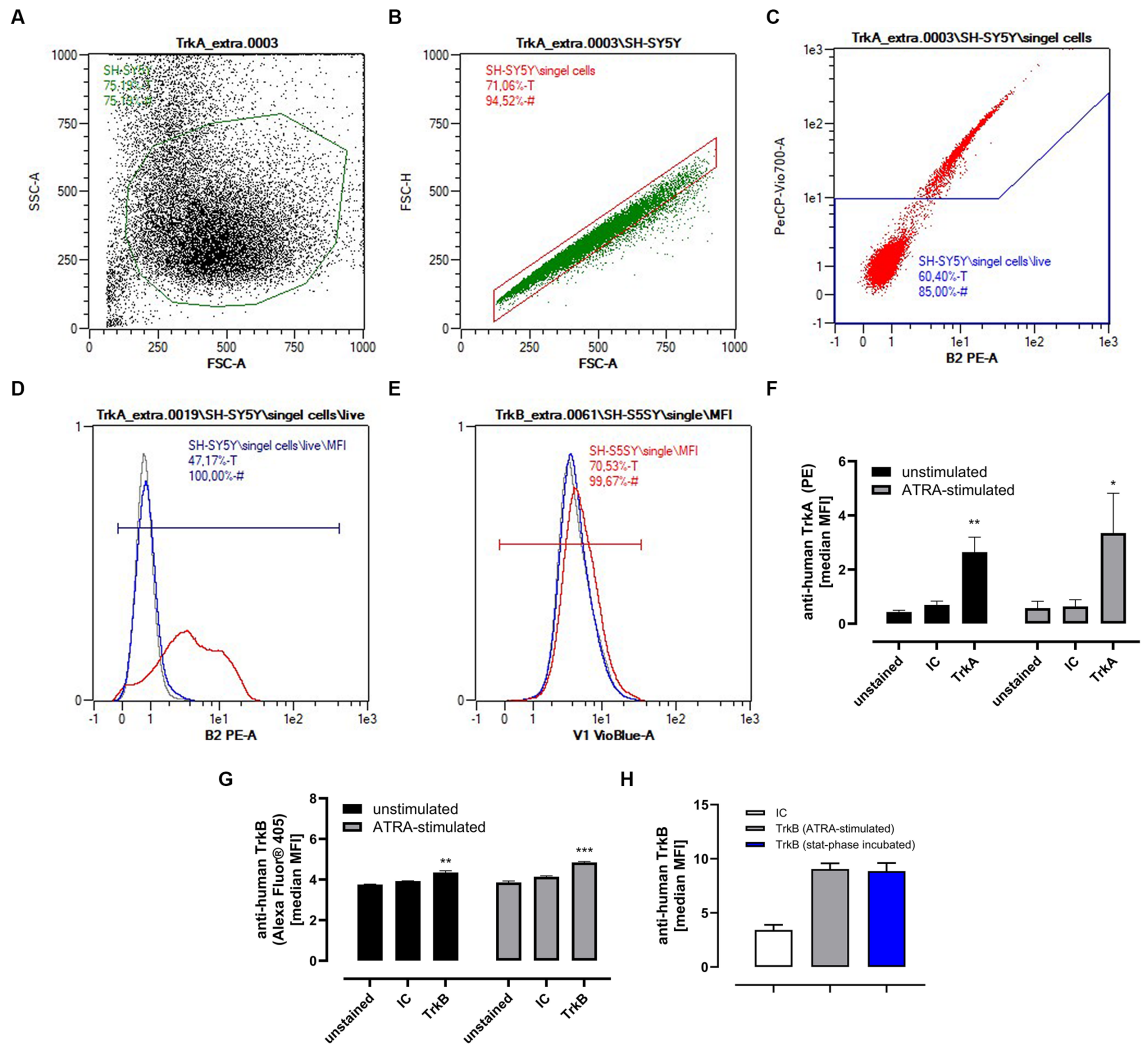
Expression of TrkA and TrkB in unstimulated SH-SY5Y and ATRA-stimulated SH-SY5Y_ATRA_ cells and the effect of metabolites from stat-growth phase supernatant from 2´-FL fermentation by *B. longum* ssp*. infantis*. Cells were incubated with or without ATRA (see Section 4.1.2) and expression of TrkA and TrkB was analyzed by flow cytometry (see Section 4.10). Therefore, after incubation, cells were treated with accutase solution to ensure surface protein integrity. After centrifugation (500 × *g*, 5 min), harvested cells (1 × 10^5^ cells/mL) were incubated with anti-human TrkA PE-conjugated, anti-human TrkB Alexa Fluor^®^ 405-conjugated monoclonal antibody or with corresponding isotype control (IC) for 10 min in the dark at room temperature. Gating strategy for analyzing TrkA and TrkB with MQ10 Analyzer for a representative staining is given in **(A–C)**. [single cells (FSC-H vs. FSC-A) **(B)**, propidium iodide-stained cells (PerCP-Vio700A vs. PE) **(C)**, representative histogram overlay of unstained (gray), isotype stained (blue line) and ATRA-stimulated Trk stained cells (red line) **(D, E)**]. Quantification of TrkA **(F)** and TrkB, **(G)** expression and influence of 2´-FL metabolite enriched media on TrkB expression **(H)**. Data are given as MFI (main fluorescence intensity) medians with CI (95%) for *n* = 3 (each in duplicates) and quantification was performed using the MACSQuant 2.13.0 software. Data analysis were performed with GraphPad Prism 10.0.2. Significant differences between IC were calculated with One-way ANOVA with multi-comparison tests. Differences were significant at *p < 0.05, **p < 0.01, and ***p < 0.001.

Because of the well-known effect of neurotrophic factors on the Trk-signaling cascade (55), we measured the protein expression of the isoforms TrkA and TrkB on SH-SY5Y and SH-SY5Y_ATRA_ cells. Both the isoforms were expressed on neuronal cells, but TrkB signaling is a well-known effect of BDNF, whereas TrkA signaling was induced by unprocessed BDNF (26, 56–58). Here (Figures 6A–H), we detected a slight but significant expression of TrkA (Figure 6F) and TrkB (Figure 6G) on unstimulated and ATRA-stimulated cells. Although BDNF has been found to increase the expression of TrkB as well as AChE (acetylcholine esterase) and ChAT activity (choline acyltransferase) (59, 60), we could not see any effect produced by the stat-phase supernatants of 2´-FL metabolized by *B. longum* ssp*. infantis* (Figure 6H).

## 3 Discussion

In the present study, we evaluated the effects of 2´-FL and Fuc, either as intact or fermented saccharides, on neuronal-like cell activity using an *in vitro* transwell co-culture model with intestinal Caco-2 cells, which reflect the intestinal cell layer in the gut, and ATRA-induced SH-SY5Y_ATRA_ cells, which are used as a model of neuronal-like cells (61–63). These SH-SY5Y_ATRA_ cells were either directly incubated with intact 2´-FL or Fuc or indirectly applied to a Caco-2 cell monolayer. In addition, 2´-FL and Fuc were fermented prior to the indirect incubation of SH-SY5Y_ATRA_ cells to assess viability, neurotransmitter release, and changes in plasma membrane and also measure mitochondrial potential. While no effects on neuronal cell activities were detected on SH-SY5Y_ATRA_ cells using intact 2´-FL or Fuc, metabolites from 2´-FL fermentation produced by *B. longum* ssp*. infantis* alone or together with *B. breve* showed an increase in BDNF secretion from SH-SY5Y_ATRA_ cells in the *in vitro* co-culture model. Although only low levels of BDNF was secreted, it was a result of enhanced vesicular release and not a result of an induction of mRNA expression, as demonstrated by the use of the L-type calcium channel blocker Verapamil (VP).

HMOs are considered to exert effects in extra-intestinal tissues such as the brain (21, 64, 65). Many studies in this connection have reported that breastfeeding is associated with higher intelligence quotient (IQ), either at school age or in adulthood (20, 66, 67). Among the fucosylated oligosaccharides, *in vitro* administered 2´-FL and Fuc were able to enhance long-term potentiation (LTP) in the rat hippocampus (68, 69). In addition, Vázquez et al. (17) reported that synaptic plasticity in rodents was enhanced after oral supplementation with 2´-FL and Wu et al. (19) have recently shown that oral intake of 2´-FL improved locomotor activity and upregulated BDNF expression in rats. In another study, no significant differences were observed between 2´-FL-supplemented rats (age 4–6 weeks post weaning) and controls in behavioral tests such as the maze tests; however, significant differences were shown at age 1 year (20).

The underlying mechanisms are poorly understood and it has been speculated that a direct effect of 2´-FL in the brain or an indirect interaction with the vagus nerve at the intestinal level is possible (17, 70, 71). Despite the data showing that 2´-FL may reach the brain via systemic circulation, we have recently shown that ^13^C enrichment in the brain tissue does not occur when mice were given ^13^C-labeled 2´-FL or Fuc via intravenous injection, indicating that none of these saccharides can cross the blood–brain barrier in mice. Furthermore, in germ-free mice orally fed with ^13^C-labeled 2´-FL, the ^13^C bolus remains in the intestinal content and was expelled via the feces, indicating that gut microbial metabolites of 2´-FL or Fuc could be responsible for the observed effects since ^13^C enrichment of brain tissue occurred when the ^13^C-2´-FL or ^13^C-Fuc bolus had reached the lower gut containing microbiota (28, 29). In this context, it is well-known that bifidobacteria were able to utilize fucosylated HMOs to produce metabolites such as short-chain fatty acids (e.g., acetate) and lactate (72–74). During breastfeeding, *B. longum* ssp. *infantis* and *B. breve* are known to regularly colonize the infant gut and express several transport proteins and glycosidases directly involved in HMO utilization according to the HMO-degrading gene cluster. For example, *B. longum* ssp. *infantis* express transport proteins and intracellular 1,2-α-L-fucosidases or 1,3-1,4-α-L-fucosidases and therefore utilize HMO by transporting them from extracellular to intracellular sites and hydrolyzing them using glycoside hydrolases (74, 75).

In the present study, using our established intestinal–neuronal transwell co-culture system, we showed that intact ^13^C_1_-labeled 2´-FL or Fuc (^13^C_1_- and ^13^C_6_-labeled) were not able to cross the polarized Caco-2 cell layer as measured by EA-IRMS. Furthermore, using media in the basal compartment, we could not detect any effects on neuronal-like cell activities in SH-SY5Y_ATRA_ cells when 2´-FL or Fuc was applied directly or indirectly via the Caco-2 layer. Based on these results, we used different *Bifidobacterium* strains to generate metabolites from 2´-FL or Fuc to further investigate their effects on neuronal-like cell activities. As expected, the bacterial strains *B. longum* ssp*. infantis* and *B. breve* alone or in combination showed different preferences with regard to 2´-FL and Fuc as carbohydrate growth substrates. *B. longum* ssp*. infantis* grew well on 2´-FL supplemented media very similar to an isomolar concentration of glucose, which was used as control. In contrast, *B. breve* preferred Fuc although to a much lower degree compared to the isomolar concentration of glucose; 2´-FL did not seem to be metabolized to support its growth. Co-incubation of *B. longum* ssp*. infantis* and *B. breve* revealed a more efficient fermentation of 2´-FL, when assessed by the pH levels (data not shown), achieved in the stationary phase of bacterial growth, suggesting an interaction of *B. longum* ssp*. infantis* and *B. breve* although a direct cross-feeding effect was not assessed.

To mimic the transport of bacterial metabolites from ^13^C-labeled 2´-FL or Fuc across the intestinal epithelium, we collected supernatants at different time points (lag-, log- and stat-growth phase) of bacterial growth, applied them to a polarized Caco-2 cell monolayer, and measured ^13^C enrichment in the basal compartment. While no ^13^C enrichment was detected in lag- and log-growth phase supernatants, ^13^C enrichment was observed in the stat-phase supernatants from *B. longum* ssp*. infantis* supplemented with 2´-FL and *B. breve* supplemented with Fuc, albeit in lower concentrations. Using supernatants from co-cultured *B. longum* ssp. *infantis* and *B. breve*, we also observed only an ^13^C enrichment in the basal compartment with supernatants from the stat-growth phase. Interestingly, we detected the release of BDNF from SH-SY5Y_ATRA_ cells only in stat-growth phase supernatants after 2´-FL fermentation from *B. longum* ssp. *infantis* containing batch cultures. Although we also observed a ^13^C enrichment in stat-growth phase supernatants from ^13^C_6_-Fuc-fermented bacterial strains, but not from ^13^C_1_-Fuc, the ^13^C enrichment was much lower than in the cultures with ^13^C_1_-2´-FL. Nevertheless, it remains speculative whether the amount or type of metabolite was responsible for the BDNF releasing effect from SH-SY5Y_ATRA_ cells. As mentioned above, *B. longum* ssp*. infantis* is able to degrade 2´-FL by several fucosidases, which may have released Fuc from 2´-FL. As the native Fuc applied directly to the differentiated cells did not produce any effect, it can be assumed that the effect was likely induced by metabolites. In this context, it has recently been shown that under anaerobic conditions Fuc was further metabolized to dihydroxyacetone-phosphate or lactate and/or 1,2-propanediole (1,2-PDO), which are intermediate productions for the generation of short chain fatty acids, i.e., lactate is a precursor of acetate and butyrate and 1,2-PDO of propionate (76). Keeping in mind that C_1_ of Fuc was ^13^C-labeled, ^13^C enrichment may rather be derived from a dihydroxyacetone phosphate metabolite than from a lactate and/or 1,2-PDO metabolite, since lactate and/or 1,2-PDO are C4,5,6-backbone molecules, whereas dihydroxyacetone phosphate results from the C1,2,3-backbone (76–78). On the other hand, it has been shown that metabolites such as lactate play an important role in LTP. A pharmacological inhibition of MCT2 (monocarboxylate transporter 2), a transporter delivering lactate to neurons, irreversibly impairs long-term memory possibly by modulating the PGC1α/FNDC5/BDNF pathway (79–81). We expected that the use of Fuc labeled either on C_1_ or C_6_ of the molecule should enable us to gain further insight into the metabolic pathways of Fuc. However, we showed that 2´-FL labeled on C_1_ of its Fuc moiety had been metabolized by *B. longum* ssp*. infantis*, but not ^13^C-labeled compounds, which were able to pass an intestinal cell layer when ^13^C-labeled Fuc was infused, labeled on either C_1_ or C_6_ of the molecule. In addition, we observed that Fuc degradation by *B. breve* led to soluble compounds containing the C_6_-atom from Fuc; the C_1_-ending of Fuc might have been completely metabolized, e.g., to CO_2_ since no ^13^C enrichment was seen in the basal compartment when ^13^C_1_-Fuc was supplemented to bacterial media. Which metabolite is responsible for the ^13^C enrichment in the basal compartment after incubation of Caco-2 cells with media from *B. longum* ssp. *infantis* or the mixture of *B. longum* ssp. *infantis* and *B. breve* needs further investigation. However, only metabolites from 2´-FL produced from *B. longum* ssp. *infantis* were able to induce secretion of BDNF in SH-SY5Y_ATRA_ cells.

As mentioned above, BDNF and its isomers are members of the neurotrophin family and have been shown to play a key role as mediators of activity-induced LTP in neuronal cells. It has been shown that BDNF mRNA expression could be induced in SH-SY5Y cells by different stimuli (54, 82) and the released BDNF protein could act at auto- and paracrine levels. As such, it is an important modulator of glutamatergic and GABAergic synapses with glutamate and GABA release through TrkB receptor signaling (27). In this context, the released BDNF binds to TrkB and activates Ras/ERK, phosphatidylinositol3-kinase/Akt and phospholipase C-g(PLC-g) signaling cascades, which in turn stimulate glutamate and GABA release as neurotransmitters (24, 25, 80). BDNF release, however, is a highly regulated process in which ER (endoplasmic reticulum)- and Golgi-associated vesicles are released either constitutively or through regulated mechanisms. The secretion via Golgi-derived vesicles requires Ca^2+^-sustained intracellular elevations and is associated with plasma membrane hyperpolarization. In addition, TrkB activation by BDNF triggers the PGC1α (peroxisome proliferator-activated receptor- γ coactivator 1-alpha) pathway, which in turn increases the expression of BDNF protein (80, 83). In our experiments, metabolites from 2´-FL produced by *Bifidobacterium* species did not affect the BDNF gene expression as confirmed by RT-qPCR but did induce a low, but significant BDNF release. Using Verapamil, a well-known L-type voltage-dependent calcium channel (VDCC) antagonist that inhibits BDNF release (84), we observed a significant, but not complete inhibition of BDNF secretion, suggesting that additional mechanisms are involved in the release of BDNF from SH-SY5Y_ATRA_ cells. This was also reported for primary neuronal cells using Verapamil as a VDCC blocker (85). Other than the observed secretion of BDNF, no further influence on choline, glutamate, or GABA release was detected, possibly due to the low levels of secreted BDNF and an unexpectedly low TrkB expression on SH-SY5Y_ATRA_ cells. Thus both the low BDNF secretion and the lack of signaling activation described above could be offered as an explanation, although TrkB receptor expression has previously been shown to be present in SH-SY5Y cells after differentiation with retinoic acid (86). In this context, it should be mentioned that several differentiation protocols for the neuroblastoma cell line SH-SY5Y into a neuronal-like cell type have been established using ATRA, B27-supplement, and BDNF, alone or in combination (59, 62, 87).

In conclusion, our ATRA/B27-supplement treatment of SH-SY5Y cells revealed a neuronal-like phenotype with increased expression levels of synaptophysin, a well-known marker of neuronal cell differentiation. Using this neuronal-like cell model, we have shown that only 2´-FL, fermented by *B. longum* ssp*. infantis* induced BDNF secretion via vesicle-releasing mechanisms. However, it remains to be determined which metabolite may be responsible for ^13^C enrichment and the effect of neuronal cell activity.

## 4 Methods and materials

### 4.1 Study design

**Figure 7:**
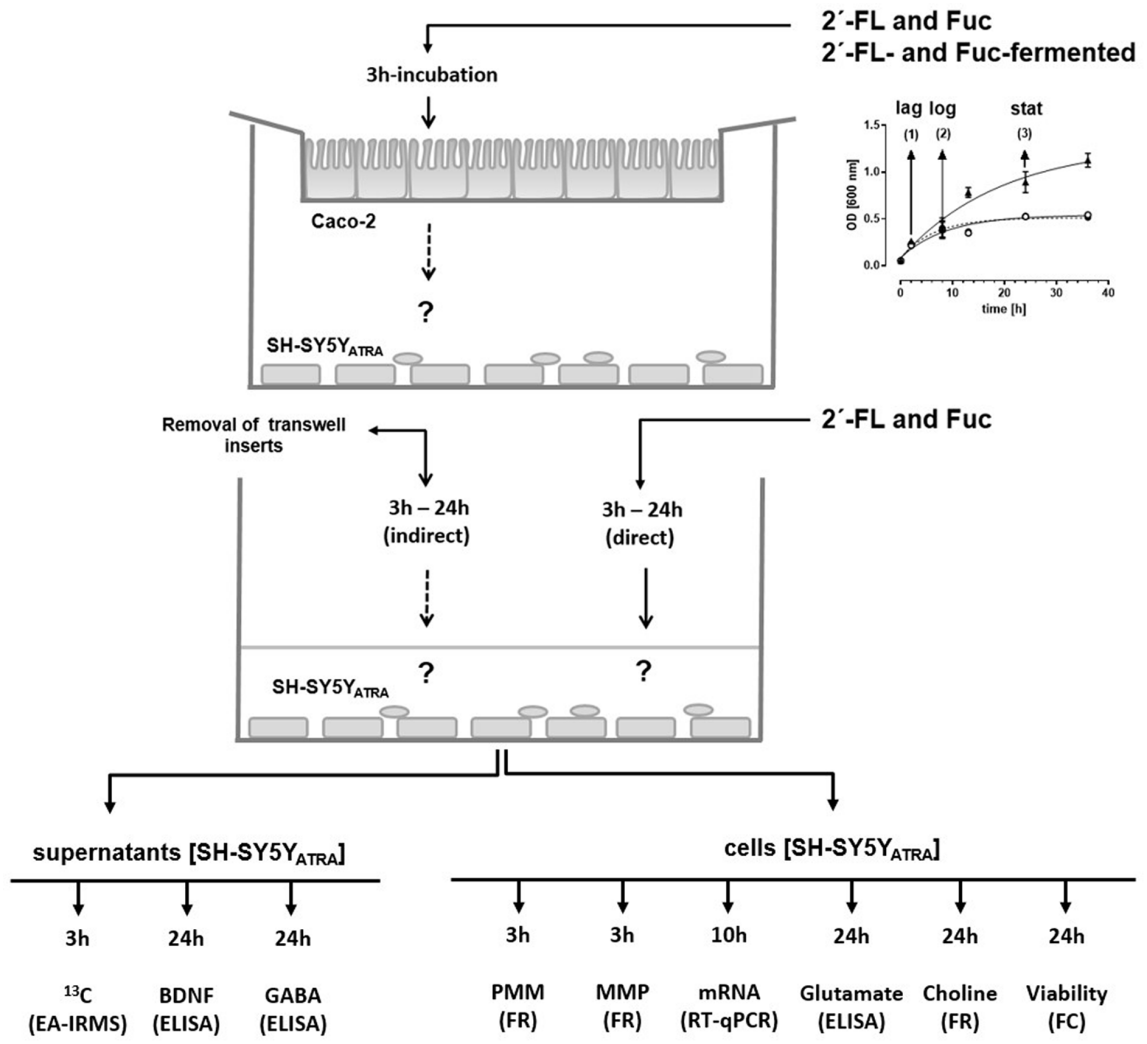
*In vitro* transwell intestinal-neuronal co-culture model. Non-fermented ^13^C-2´-FL (5 mM) and ^13^C-Fuc (5 mM) as well as ^13^C-2´-FL or ^13^C-Fuc fermented by *B. longum* ssp. *infantis, B. breve,* and *B. longum* ssp. *infantis /B. breve,* collected at the lag-, log-, and stat-growth phase, were applied to the transwell inserts cultivated with 22-day differentiated Caco-2 cells (“indirect incubation”). After a 3 h of incubation, Caco-2 inserts were removed and 10-day ATRA-differentiated SH-SY5Y cells (SH-SY5Y_ATRA_) were used immediately or incubated for the times indicated to measure neuronal cell activities. For “direct” incubation, ^13^C-2´-FL (0.5 mM) and ^13^C-Fuc (0.5 mM) were incubated with SH-SY5Y_ATRA_ cells. After indicated incubation times of SH-SY5Y_ATRA_ cells, basal compartments (supernatants) were collected to measure ^13^C enrichment by EA-IRMS, BDNF, and GABA concentrations by ELISA. Cells were used to measure membrane potential [plasma (PMM) and mitochondrial (MMP)] and choline levels by fluorescence kits using a fluorescence reader (FR), mRNA-expression of BDNF by real-time quantitative PCR (RT-qPCR), glutamate by ELISA, and viability by flow cytometry (FC).

In order to investigate the effects of non-fermented and fermented 2´-FL and Fuc on neuronal cell activity markers, we developed an *in vitro* transwell co-culture model in which human intestinal epithelial cells (Caco-2) and ATRA-differentiated SH-SY5Y neuronal-like cells (SH-SY5Y_ATRA_) were able to impact each other (Figure 7) similar to our previously published *in vitro* epithelial-endothelial co-culture model (88). In order to mimic the absorption and metabolization sites in the intestine, Caco-2 cells were grown on semipermeable transwell filters over 22 days to differentiate and develop an enterocyte-like phenotype. After differentiation, transwell filters were inserted into a 24-well cavity where SH-SY5Y_ATRA_ cells were cultivated at the bottom of the cavity. The upper compartment (transwell insert) with epithelial cells was exposed with non-fermented and fermented 2´-FL and Fuc for 3 h (indirect incubation). Thereafter, inserts were removed and basal media (supernatants) as well as SH-SY5Y_ATRA_ cells were used immediately or after indicated times in order to determine neuronal cell activity markers. Direct incubation was done using intact 2´-FL and Fuc directly on SH-SY5Y_ATRA_ cells.

#### 4.1.1 Culturing intestinal Caco-2 cells

The human intestinal epithelial cell line Caco-2 (HTB37™) was derived from colon adenocarcinoma cells obtained from ATCC (Manassas, Virginia, United States). The cells were routinely grown in 75 cm^2^ culture flasks using Dulbecco’s Eagle’s Minimum Essential Medium (DMEM) at pH 7.4 with 1% non-essential amino acids (NEAA), 1% sodium pyruvate, and 10% fetal calf serum (FCS, Invitrogen, Germany). Cells were maintained in a humidified atmosphere of 5% CO_2_ in air at 37°C. Stock passages were sub-cultured every 4 days until reaching 70–80% confluence. For incubation studies, pre-confluent cells were trypsinized with a 0.25% (w/v) trypsin/0.53 mM EDTA solution (Invitrogen, Darmstadt, Germany) and 1 × 10^4^ cells per 0.5 mL^−1^ were seeded onto a 24-well transwell-insert with a polycarbonate membrane (0.4 μm pore size, Greiner-Bio-One GmbH, Frickenhausen, Germany) and placed in a 24-well cavity. Cells were allowed to grow to confluence (2 days) with DMEM (20% FCS) and thereafter to differentiate to absorptive enterocytes within 22 days. The culture medium was changed every 2–3 days at the apical (0.5 mL) and basolateral sides (1.5 mL). For incubation experiments at day 22, the transepithelial electrical resistance (TEER), a marker of the integrity of polarized epithelial cell monolayers, was determined before and after the experiments by using a Millicell^®^ ERS volt-ohmmeter (Millipore Corporation, Bedford, MA, United States). TEER readings were taken at 37°C after equilibrium with the incubation media. A TEER value ≥800 Ohm × cm^2^ was used as an indicator for an intact epithelial layer suitable to be used for incubation studies.

#### 4.1.2 Culturing neuroblastoma SH-SY5Y cells

The human neuroblastoma cell line SH-SY5Y (ACC209) was obtained from DSMZ (German Collection of Microorganisms and Cell Cultures GmbH, Braunschweig, Germany). Cells were cultured (5% CO_2_; 37°C) in Ham’s F12/DMEM (1:1; with GlutaMAX™, sodium bicarbonate and sodium pyruvate) and supplemented with 15% FCS (Invitrogen GmbH, Karlsruhe, Germany). Cells were routinely sub-cultured splitting sub-confluent cultures (70–80%) 1:10 with 0.5% (w/v) trypsin/0.25 mM EDTA solution (Invitrogen GmbH, Karlsruhe, Germany). Cells grow as undifferentiated, continuously proliferating cells and include both adherent and floating cells. For sub-cultivation, one third of the supernatant with floating cells was collected, centrifugated (500 × *g*, 5 min at RT) and taken up in fresh complete media. Pre-confluent adherent cells were trypsinized with a 0.5% (w/v) Trypsin/0.25 mM EDTA solution and after centrifugation (500 × *g*, 10 min at room temperature), 1 × 10^5^ cells ml^−1^ were seeded into a new culture flask and combined with the pre-collected floating cells. For incubation studies (direct or indirect (co-culture system)), adherent and floating SH-SY5Y cells were cultured in serum-reduced medium (2.5% FCS) containing 10 μM all-*trans-*retinoid-acid (ATRA) (Merck, Darmstadt, Germany) on a 24-well-plate and allowed to differentiate within 8 days according to Teppola et al. (89) and Al-Maswary et al. (90) with slight modification. After 24 h of sub-culturing, serum reduced medium was replaced with a medium containing B-27™ supplement (ThermoFischer Scientific, Darmstadt, Germany) and 10 μM ATRA to promote differentiation into a neuronal-like phenotype. Stock solution of ATRA was diluted in 96% ethanol and the final ethanol concentration did not exceed 0.1% in cell culture medium. Control cells were treated with vehicle (0.1% ethanol). This treatment was replaced every 3 days to replenish ATRA in culture media and, after the differentiation protocol, SH-SY5Y_ATRA_ differentiation was confirmed by flow cytometry with SYP as a well-known neuronal marker (51, 52). Therefore, after detachment of SH-SY5Y_ATRA_ cells with accutase solution (PromoCell GmbH, Heidelberg Germany), cells were centrifugated (500 × *g*, 5 min at RT) and stained according the manufacturer’s instructions with slight modifications. Centrifuged cells were resuspended in 100 μL MACS buffer (Miltenyi Biotec B.V. & Co. KG, Bergisch-Gladbach, Germany) and were fixed for 20 min in darkness with 150 μL Cyto Fast Perm FIX buffer (BioLegend^®^, Amsterdam, Netherland). After washing, step cells were permeabilized and stained with 98 μL Cyto Fast Perm solution with 2 μL anti-human Synaptophysin-APC REAfinity antibody (Miltenyi Biotec B.V. & Co. KG) for 10 min at room temperature (Table 1). Unbound antibodies were removed by washing the cells in 1 mL running buffer (Miltenyi Biotec B.V. & Co. KG). After centrifugation (500 × *g*, 10 min), cells were resuspended in 200 μL of MACS running buffer for final flow cytometry analysis in R1-APC channel. Cell gating strategy and quantification (Figures 1A–D) were performed using the MACSQuant 2.13.0 software (Miltenyi Biotec B.V. & Co. KG) by comparing the median fluorescence intensities (MFI) of unstained, isotype stained and SYP-stained cells with at least *n* = 3 (each done in duplicates).

**Table 1:** Antibodies for Synaptophysin staining of SH-SY5Y and SH-SY5Y_ATRA_ cells.

Target	Primary recombinant antibodies (Ab)	Fluorochrome	Dilution	Incubation time	MQ10 channel
Synaptophysin (SYP), clone REA1121	REAfinity^®^	APC	1:50	10 min	R1 (APC)
Isotype control for SYP	Recombinant human IgG1	APC	1:50	10 min	R1 (APC)

#### 4.1.3 Co-culturing intestinal and neuronal-like cells with a transwell system

In order to investigate the effects of non-fermented and fermented 2´-FL and Fuc on neuronal-like cells, we developed an *in vitro* transwell co-culture system with human intestinal epithelial cells (Caco-2) and SH-SY5Y_ATRA_ cells (Figure 7). According to the experimental setting, differentiated Caco-2 cells on transwell filter inserts were placed onto a 24-well plate, where SH-SY5Y_ATRA_ cells had been cultured as described above. In a first set of experiments, non-fermented 2´-FL and Fuc were exposed directly to SH-SY5Y_ATRA_ cells in order to evaluate the direct effect on neural activity markers. In a second set of experiments, non-fermented and fermented 2´-FL and Fuc were exposed to the upper compartment with Caco-2 cells on transwell filters (indirect incubation) (see Section 4.1).

#### 4.1.4 Isotope-labeled 2´-FL and Fuc

Stable isotope labeled 2´-FL containing the C-atom 1 in the fucose ring as ^13^C ([1 -^13^C_1_]-2´-FL (^13^C-2’FL)) was obtained from ELICITYL (Crolles, France). In addition, we used L-Fuc, which was ^13^C-labeled either at C_1_ [^13^C_1_-Fuc] or C_6_ [^13^C_6_-Fuc] also with a ^13^C enrichment of 99% (ELICITYL). Both were used either at a concentration of 0.5 mM for direct incubation or 5 mM for the fermentation studies (indirect incubation).

#### 4.1.5 Bacterial fermentation of 2´-FL and Fuc

2´-FL or Fuc metabolites were generated by batch cultivation of 2´-FL or Fuc with *B. longum* ssp. *infantis* (DSM 20088) obtained from the German Collection of Microorganisms and Cell Cultures GmbH (DSMZ, Braunschweig, Germany) and with *B. breve* (DSM 20213) as a gift from Prof. Dr. Sylvia Schnell (Department of Applied Microbiology, Justus-Liebig University Giessen, Germany). Rehydration of freeze-dried bacterial strains and − 80°C stock cultures were done according to the manufacturer’s instructions*. B. longum* ssp*. infantis* and *B. breve* were routinely cultured at 37°C in ‘Bifidobacterium medium’ containing 10 g/L casein peptone (tryptic digest), 10 g/L glucose, 5 g/L yeast extract, 5 g/L meat extract, 5 g/L bacto soytone, 2 g/L K_2_HPO_4_, 0.2 g/L MgSO_4_·7H_2_O, 0.05 g/L MnSO_4_·H_2_O, 1 mL/L Tween80, 5 g/L NaCl, 40 mL salt solution (0.25 g/L CaCl_2_·2 H_2_O, 0.5 g/L MgSO_4_·7H_2_O, 1.0 g/L K_2_HPO_4_, 1.0 g/L KH_2_PO_4_, 10.0 g/L NaHCO_3_, 2.0 g/L NaCl), and 4 mL/L resazurin (250 mg/L) dissolved in distilled water and autoclaved at 121°C for 40 min. Thereafter, the medium was left within the autoclave until reaching 98°C and was then further cooled down under oxygen-free gas (10% CO_2_, 80% N_2_, and 10% H_2_) to avoid redissolving of oxygen. After autoclavation, pH was adjusted to pH 6.8 using NaOH (8 M) and supplemented with sterile filtered 0.5 g/L l-cysteine hydrochloride. Then, the medium was dispensed into Hungate anaerobic culture tubes under gas. Both strains were grown in independent triplicates under anaerobic condition at 37°C and growth was assayed by the determination of an increase in optical density (OD) at 600 nm using Shimadzu UV 1001 spectrophotometer (Shimadzu GmbH, Duisburg, Germany).

For incubation studies, corresponding bacterial growth media was prepared glucose-free and substrate utilization was determined by adding sterilized glucose or ^13^C-labeled compounds to glucose-free medium. To obtain working cultures, cultivated stock cultures were incubated three times in carbohydrate-reduced medium to adapt microorganisms to the incubation media. After inoculation with bacterial suspensions for *in vitro* co-culture experiments, samples were taken at three different time points: the lag-growth phase, the logarithmic growth phase (log-growth phase), and stationary growth phase (stat-growth phase). After centrifugation (5 min, 13,000 rpm), the bacteria-free culture media were filtered through a 0.2 μm PES Whatman syringe filters (FisherScientific, Schwerte, Germany) and were used immediately for functional assays or stored at −80°C until for further analyses.

### 4.2 Determination of ^13^C enrichment by elemental analysis isotope mass spectrometer

To analyze cell culture samples for ^13^C enrichment, 0.15 mg liquid samples (apical cell culture samples) were weighted into tin capsules containing 5 mg of acid-washed Chromosorb W (IVA Analysentechnik e.K., Meerbusch, Germany). Triplicate samples were subjected to Elemental Analysis Isotope Ratio Mass Spectrometry (EA-IRMS) as described previously (28). Measurements and calculations were performed using the IonVantage Software v1.7 in combination with Ionos v4.2; both software applications were obtained from Elementar UK (Stockport, United Kingdom). Results are expressed as δ^13^C enrichment [‰] with VPDB being the international standard obtained from the International Atomic Energy Agency (IAEA, Vienna, Austria).

### 4.3 Viability

A subset of cultured SH-SY5Y_ATRA_ cells was used for measuring cell viability to ensure the viability of cells during co-cultivation by using the ViaCount™-assay (Luminex BV, MV ´s-Hertogenbosch, Netherland). Thus, cells were trypsinized using a 0.5% (w/v) trypsin/0.25 mM EDTA solution (Invitrogen) after 24 h incubation. After centrifugation (500 × *g*, 10 min), the pelleted cells were suspended in 500 μL of PBS. Following this, 20 μL of the cell solution was incubated with 480 μL ViaCount-Reagent™ and incubated for 10 min in the dark at 37°C. Immediately after Live/Dead-staining, cells were measured by flow cytometry on the Guava EasyCyte Mini Flow Cytometer (Guava Technologies, Merck Millipore, Darmstadt, Germany). Viability was expressed as % viable cells of totals with the Guava^®^ software (*n* = 3, each done in duplicates). Further, 480 μL of the cell solution were used for glutamate detection in cell lysates (see Section 4.4).

### 4.4 Detection of neurotransmitters (BDNF, GABA, and glutamate)

The secretion of the neurotransmitter BDNF and GABA were measured in the supernatant of 24 h- stimulated SH-SY5Y_ATRA_ cells using BDNF Quantikine™ ELISA Kit (R&D, Heidelberg, Germany) and ELISA kit for GABA (Abcam, Rozenburg, Germany). Glutamate as a precursor for GABA was measured in SH-SY5Y_ATRA_ cell lysates according to the manufacturer’s instructions with the Glutamate ELISA Kit (Abcam). Briefly, after incubation of the SH-SY5Y_ATRA_, supernatants were collected and stored at –20°C until analysis for BDNF and GABA. For glutamate quantification, 480 μL of trypsinized SH-SY5Y_ATRA_ cells (see Section 4.3) was used immediately and washed twice with PBS and lysed with lysis buffer for 20 min. Afterward, lysed cells were centrifugated (500 × *g*, 10 min) and supernatant was collected and used according to the manufacturer’s instructions (Abcam). BDNF and GABA concentrations were measured at 450 nm and glutamate concentration were measured at 405 nm using the DigiScan microplate reader (Asys, Eugendorf, Austria). The BDNF and GABA concentrations were expressed as pg./mL and glutamate concentrations were expressed as μg/mL with *n* = 3 (each done in duplicates).

### 4.5 Choline levels

The total choline levels (free choline and acetylcholine) in SH-SY5Y_ATRA_ cells were measured using the fluorometric Choline/Acetylcholine Assay Kit (Abcam) in freshly prepared samples according to the manufacturer’s instructions. Briefly, after direct or indirect incubation of SH-SY5Y_ATRA_ cells, cells were harvested by trypsinization (0.5% (w/v) trypsin/0.25 mM EDTA solution) and were washed twice with ice-cold PBS (Invitrogen GmbH). The cell pellet was resuspended in 500 μL choline assay buffer and homogenized by pipetting up and down ten times and leaving the cells for 10 min on ice. After centrifugation (500 × *g*, 5 min, 4°C), the supernatant was collected and the assay was done according to the manufacturer’s instructions. Acetylcholine was converted to choline by adding acetylcholinesterase and free choline was oxidized via the intermediate betaine aldehyde to betaine. The reaction generates products, which react with the choline probe to generate a fluorescence signal (Ex/Em 535/585 nm). Total choline concentrations were measured in black-clear microtiter plates (Greiner Bio-One GmbH, Frickenhausen, Germany) using the Ascent microplate fluorescence reader (Thermo Fisher Scientific, Germany). Fluorescence values (RFU) were finally expressed as % of controls with *n* = 3 (each done in duplicates).

### 4.6 Plasma membrane potential

Plasma membrane potential (PMP) was measured with the fluorogenic membrane Assay Kit (Abcam) according to the manufacturer’s instructions. Briefly, after “direct” incubation of SH-SY5Y_ATRA_ cells with 2´-FL and Fuc for 3 h or “indirect” incubation with basal media from transwell studies, the medium was replaced with 150 μL assay buffer (1:10) including 2 μL MP sensor dye. After 30 min of incubation in a 5% CO_2_ incubator, fluorescence intensity (Ex/Em 535/585 nm) was measured using the Ascent microplate fluorescence reader (Thermo Fisher Scientific). Fluorescence values (RFU) were finally expressed as % of controls with *n* = 3 (each done in duplicates).

### 4.7 Mitochondrial membrane potential

Mitochondrial membrane potential (MMP) was measured with the fluorogenic JC-1 Assay Kit (Abcam) according to Sakamuru et al. (91). Briefly, after `direct´ incubation of ATRA-differentiated SH-SY5Y cells with 2´-FL and Fuc over 3 h or `indirect´ incubation with basal media from transwell studies, the medium was replaced with 200 μL HBSS buffer including 5 μM JC-1 solution (5,5′,6,6′-tetrachloro-1,1′, 3,3′-tetraethylbenzimidazol-carbocyanine iodide). After 30 min of incubation in a 5% CO_2_ incubator, and after two washing steps with HBSS, fluorescence intensity (Ex/Em 485/535 nm) was measured in using the Ascent fluorescence reader (Thermo Fisher Scientific). Fluorescence values (RFU) were finally expressed as % of controls with n = 3 (each done in duplicates).

### 4.8 Determination of mRNA expression of BDNF by RT-qPCR

mRNA was isolated directly from SH-SY5Y_ATRA_ derived from the incubation experiments using the Dynabeads mRNA DIRECT™ kit (Invitrogen GmbH) according to the manufacturer’s instructions. After the isolation of mRNA, cDNA synthesis was carried out with the iScript cDNA synthesis kit using the C1000 Touch Thermal cycler (Bio-Rad Laboratories GmbH, Feldkirchen, Germany) in a reaction volume of 10 μL containing 20 ng mRNA with iScript reaction mix (5×), iScript Reverse Transcriptase and nuclease-free water (Bio-Rad Laboratories GmbH). Samples were incubated at 25°C for 5 min, followed by an incubation at 46°C for 20 min, and inactivation at 95°C for 1 min. Amplification of target genes (BDNF and ß-Actin (ACTB)) was measured using the C1000 Touch Thermal cycler (Bio-Rad Laboratories GmbH, Feldkirchen, Germany) with gene-specific primers/probe sets labeled with FAM (BDNF-PrimePCR™ Probe Assay) or HEX (ACTB-PrimePCR™ Probe Assay). Amplification were done with 2 μL cDNA in a reaction volume of 20 μL containing iTaq Universal Probes Supermix (2×), PrimePCR™ Probe Assay (Bio-Rad Laboratories GmbH), and water in a two-step amplification with 3 min of initial denaturation at 95°C, followed by 45 cycles of 5 s at 95°C and 30 s at 60°C. The relative expression level was measured using the ΔΔ C_T_-method, in which ΔC_T_ was calculated by subtracting the C_T_ value of ACTB from the specific C_T_ value of the BDNF. ΔΔ C_T_ was obtained by subtracting the Δ C_T_ of each experimental sample by the Δ C_T_ of a positive control (92, 93). Expression levels were given as % of control with *n* = 3 (each done in duplicates).

### 4.9 Inhibition of BDNF-secretion by calcium channel blocker verapamil

Inhibition of BNDF secretion was measured using BDNF Quantikine™ ELISA Kit (R&D GmbH, Heidelberg, Germany) as described above (see Section 4.4). Briefly, before incubation of SH-SY5YATRA cells with supernatants from batch culture collection at stat-growth phase, cells were washed twice with pre-warmed HBSS and were then pre-incubated for 30 min with 7.5 μM or without verapamil (> 99%; Calbiochem^®^, Merck, Germany). Afterward, the cells were washed twice with medium and finally incubated with the metabolite enriched media. Briefly, after 24 h of incubation, supernatants were collected and stored at –20°C until analysis for BDNF. Data are given as pg./mL with n = 3 (each in duplicate).

### 4.10 Flow cytometry analysis for TrkB expression

For cytometry analysis, cells were treated with accutase solution (Promocell, Heidelberg, Germany) for 10 min to ensure surface protein integrity. For TrkA staining cells were centrifugated (500 × *g*, 10 min) and stained immediately with anti-human TrkA PE-conjugated REAfinity antibody (Miltenyi Biotec B.V. & Co. KG) for 10 min in the dark (Table 2). For TrkB staining, cells were incubated after centrifugation with F_C_-blocking reagent (Miltenyi Biotec B.V. & Co. KG) for 15 min. Then, cells were incubated with mouse anti-human TrkB Alexa Fluor^®^ 405-conjugated monoclonal antibody or Alexa Fluor^®^ 405-conjugated isotype control (IgG_1_) antibody (R&D GmbH) for 10 min in the dark at room temperature. Unbound antibodies were removed by washing the cells in 1 mL running buffer (Miltenyi Biotec B.V. & Co. KG) and after centrifugation (500 × *g*, 10 min), cells were resuspended in 200 μL of running buffer for final flow cytometry analysis. Cell gating strategy and quantification (see Figure 6) were performed using the MACSQuant 2.13.0 software (Miltenyi Biotec B.V. & Co. KG) by comparing the median fluorescence intensities (MFI) of unstained, isotype stained, and Trk-stained cells with n = 3 (each done in duplicates).

**Table 2:** Antibodies for Trk staining of SH-SY5Y and SH-SY5Y_ATRA_ cell.

Target	Primary recombinant antibodies (Ab)	Fluorochrome	Dilution	Incubation time	MQ10 channel
TrkA	REAfinity^®^	PE	1:50	10 min	B2 (PE)
TrkB	Monoclonal anti-human	Alexa Flour^®^ 405	1:50	10 min	V1 (Vioblue)
Isotype control	Monoclonal mouse IgG1	Alexa Flour^®^ 405	1:50	10 min	V1 (Vioblue)

### 4.11 Statistical analysis

Statistical analyses were carried out using GraphPad Prism 10.0.2 (GraphPad Software, MA, United States) As indicated, data were analyzed by one-way ANOVA and multicomparison test or *t*-test. Differences were considered significant at *p < 0.05, **p < 0.01, and ***p < 0.001.

## Data availability statement

The original contributions presented in the study are included in the article/Supplementary material, further inquiries can be directed to the corresponding author.

## Ethics statement

Ethical approval was not required for the studies on humans in accordance with the local legislation and institutional requirements because only commercially available established cell lines were used.

## Author contributions

SK: Conceptualization, Data curation, Formal analysis, Investigation, Methodology, Validation, Visualization, Writing – original draft, Writing – review & editing. CK: Conceptualization, Funding acquisition, Resources, Writing – review & editing. CB: Investigation, Writing – review & editing. DH: Writing – review & editing. SM: Writing – review & editing. RB: Conceptualization, Writing – review & editing. SR: Conceptualization, Funding acquisition, Methodology, Project administration, Resources, Supervision, Validation, Writing – original draft, Writing – review & editing.
